# Discrimination between Alzheimer’s Disease and Late Onset Bipolar Disorder Using Multivariate Analysis

**DOI:** 10.3389/fnagi.2015.00231

**Published:** 2015-12-14

**Authors:** Ariadna Besga, Itxaso Gonzalez, Enrique Echeburua, Alexandre Savio, Borja Ayerdi, Darya Chyzhyk, Jose L. M. Madrigal, Juan C. Leza, Manuel Graña, Ana Maria Gonzalez-Pinto

**Affiliations:** ^1^Department of Psychiatry, University Hospital of Alava-Santiago, Vitoria, Spain; ^2^Centre for Biomedical Research Network on Mental Health (CIBERSAM), Madrid, Spain; ^3^School of Medicine, University of the Basque Country, Vitoria, Spain; ^4^School of Psychology, University of the Basque Country, San Sebastian, Spain; ^5^Computational Intelligence Group (GIC), University of the Basque Country, San Sebastian, Spain; ^6^ENGINE Centre, Wrocław University of Technology, Wrocław, Poland; ^7^Department of Computer and Information Science and Engineering, University of Florida, Gainesville, FL, USA; ^8^Department of Pharmacology, Faculty of Medicine, University Complutense and IIS Hospital 12 de Octubre, Madrid, Spain; ^9^Asociacion de Ciencias de la Programacion Python San Sebastian (ACPySS), San Sebastian, Spain

**Keywords:** late onset bipolar disorder, Alzheimer’s disease, computer-aided diagnosis, clinical, neuropsychological, and blood, biomarkers, pharmacological

## Abstract

**Background:**

Late onset bipolar disorder (LOBD) is often difficult to distinguish from degenerative dementias, such as Alzheimer disease (AD), due to comorbidities and common cognitive symptoms. Moreover, LOBD prevalence in the elder population is not negligible and it is increasing. Both pathologies share pathophysiological neuroinflammation features. Improvements in differential diagnosis of LOBD and AD will help to select the best personalized treatment.

**Objective:**

The aim of this study is to assess the relative significance of clinical observations, neuropsychological tests, and specific blood plasma biomarkers (inflammatory and neurotrophic), separately and combined, in the differential diagnosis of LOBD versus AD. It was carried out evaluating the accuracy achieved by classification-based computer-aided diagnosis (CAD) systems based on these variables.

**Materials:**

A sample of healthy controls (HC) (*n* = 26), AD patients (*n* = 37), and LOBD patients (*n* = 32) was recruited at the Alava University Hospital. Clinical observations, neuropsychological tests, and plasma biomarkers were measured at recruitment time.

**Methods:**

We applied multivariate machine learning classification methods to discriminate subjects from HC, AD, and LOBD populations in the study. We analyzed, for each classification contrast, feature sets combining clinical observations, neuropsychological measures, and biological markers, including inflammation biomarkers. Furthermore, we analyzed reduced feature sets containing variables with significative differences determined by a Welch’s *t*-test. Furthermore, a battery of classifier architectures were applied, encompassing linear and non-linear Support Vector Machines (SVM), Random Forests (RF), Classification and regression trees (CART), and their performance was evaluated in a leave-one-out (LOO) cross-validation scheme. *Post hoc* analysis of Gini index in CART classifiers provided a measure of each variable importance.

**Results:**

Welch’s *t*-test found one biomarker (Malondialdehyde) with significative differences (*p* < 0.001) in LOBD vs. AD contrast. Classification results with the best features are as follows: discrimination of HC vs. AD patients reaches accuracy 97.21% and AUC 98.17%. Discrimination of LOBD vs. AD patients reaches accuracy 90.26% and AUC 89.57%. Discrimination of HC vs LOBD patients achieves accuracy 95.76% and AUC 88.46%.

**Conclusion:**

It is feasible to build CAD systems for differential diagnosis of LOBD and AD on the basis of a reduced set of clinical variables. Clinical observations provide the greatest discrimination. Neuropsychological tests are improved by the addition of biomarkers, and both contribute significantly to improve the overall predictive performance.

## 1 Introduction

Bipolar disorder (BD) is a chronic mood disorder associated with cognitive, affective, and functional impairment, often appearing at youth (around age 20 years), or even earlier, whose age of onset may be determined by environmental conditions (Bauer et al., 2014b, 2015a,b; Martinez-Cengotitabengoa et al., 2014). Dementia syndrome arising after a lifetime history of bipolarity (Lebert et al., 2008; Ng et al., 2008) does not match the criteria of Alzheimer’s disease (AD) (Forcada et al., 2014). On the other hand, late onset (i.e., age > 50 years) of BD (LOBD) (Depp and Jeste, 2004; Prabhakar and Balon, 2010; Besga et al., 2011; Carlino et al., 2013; Po-Han et al., 2015) may be difficult to differentiate from behavioral impairment associated with Alzheimer’s disease (AD), because of overlapping symptoms and neuropathology. Though AD and LOBD are considered distinct and unrelated clinical entities, there is a trend in recent years to question whether there is a link between both disorders based on the overlapping symptoms and the increased successful use of well-established BD treatments, i.e., Lithium, to treat dementia (Takeshi et al., 2006).

### 1.1 Common Traits Between LOBD and AD

Most studies focus on the differences and commonalities between BD and schizophrenia (García-Bueno et al., 2014), and depression (Azorin et al., 2015); however, some recent studies report comparisons between BD and AD patients (Berridge, 2013) due to either late onset or BD aging population. Inflammation and oxidative stress have been found as common pathophysiological processes underlying AD (Akiyama et al., 2000; Kamer et al., 2008; Sardi et al., 2011) and LOBD (Goldstein et al., 2009; Konradi et al., 2012; Leboyer et al., 2012; Lee et al., 2013; Bauer et al., 2014a; Hope et al., 2015), as well as many other neuropsychological illness, such as depression and mania (Brydon et al., 2009; Dickerson et al., 2013; Castanon et al., 2014; Singhal et al., 2014). These disorders seem to be epigenetically linked to decrease transcriptional activity. It has been reported that the frontal cortex of both LOBD and AD patients exhibits an altered epigenetic regulation related to neuroinflammation, synaptic integrity, and neuroprotection (Rao et al., 2012). Oxidative stress contributes to the pathogenesis of both diseases through similar mechanisms of neuroinflammation, excitotoxicity, and upregulated brain metabolism (Rao et al., 2010, 2011). Mood and cognition impairment are considered core problems in LOBD and AD, respectively. However, in recent years, clinical features of AD, as well as cognitive deficits in LOBD, have received more attention (Ng et al., 2008). Increased agitation and aggression with cognitive and independence decline in AD can be easily confused with LOBD (Zahodne et al., 2015). The following psychiatric symptoms have been reported in AD in common with the profile observed in LOBD: agitation, euphoria, disinhibition overactivity without agitation, aggression, affective liability, dysphoria, apathy, impaired self-regulation, and psychosis (Albert and Blacker, 2006).

### 1.2 Description of the Study

The study was registered as an observation trial^1^ in the ISRCTN registry. It involved nearly one hundred subjects of age at recruitment above 64 years, including healthy controls and patients with diagnosis of AD or BD. The study included neuroimage data, neuropsychological tests, and blood sample biomarkers. Classification results based on neuroimage data have been reported elsewhere (Graña et al., 2011; Besga et al., 2012), showing that features extracted from fractional anisotropy coefficients of diffusion-weighted images provided very high classification performance between LOBD and AD patients. The hypothesis explored in the work reported here is the feasibility of AD and LOBD discrimination using multivariate machine learning-based computer-aided diagnosis (CAD) tools on a reduced set of clinical, cognitive, and biological biomarker variables. We evaluated the classification performance achieved using various feature sets composed of combinations of variable categories, as well as a feature selection based on the Welch’s *t*-test. Predictive CAD systems have been proposed to improve diagnostic accuracy complementing the neuropsychological assessments carried out by expert clinicians (Sigut et al., 2007; Graña et al., 2011; Savio et al., 2011; Westman et al., 2011; Termenon et al., 2013). Accurate diagnosis is crucial to mitigate negative effects of inappropriate treatments.

## 2 Materials and Methods

Multivariate analysis methods (Westman et al., 2011) allow to assess the joint significance of groups of biomarker measures. They have been successfully applied to large dimensionality neuroimage data (Fung and Stoeckel, 2007; Salas-Gonzalez et al., 2009). Achieved cross-validation classification accuracy, sensitivity, specificity, and area under the ROC curve (AUC) provide the significance value for each combination of variables considered as classification features. Approaches applying feature extraction by functional transformations of the data, such as orthogonal partial least squares (Ramirez et al., 2010; Westman et al., 2011), have achieved high classification performances. However, these transformations do not allow to back-project the contribution of each variable to classification success. In order to reason about disease mechanisms and quality of biomarkers, we follow a feature selection approach, where variables are selected according to their expected contribution to the classification success. Moreover, we report variable importance computed on the basis of the contribution of each variable to the construction of a specific CART classifier, as well as the Welch’s *t*-test statistical significance of variable differences between groups.

### 2.1 Subjects

Patients included in the study were referred to the psychiatric unit at Alava University Hospital, Vitoria, from its catchment recruitment area for clinical assessment of memory complaints. The BD patients were in the euthymic state. No patient has previous BD diagnosis. These patients were all living in the community. Selected subjects underwent a standard protocol, including clinical, cognitive, and neuropsychological evaluations. Ninety-five elderly subjects were included in the present study; Table 1 presents demographic details of the cohort. Sample size was conditioned by the availability of funding to carry out MRI neuroimaging and biochemical tests. Reports on neuroimage results are published elsewhere (Besga et al., 2011). The LOBD group fulfills the DSM IV criteria and the AD group fulfills the NINDS-ADRDA criteria for probable AD. Subjects with psychiatric disorders (i.e., major depression) or other conditions (i.e., brain tumors) were not considered for this study. The exclusion criteria were ongoing infections, fever, allergies, or the presence of other serious medical conditions (autoimmune, cardiac, pulmonary, endocrine, and chronic infectious diseases, and neoplasms). Neither the patients nor the healthy control subjects were receiving immunosuppressive drugs or vaccinations for at least 6 months prior to inclusion in the study or anti-inflammatory analgesics 2 days prior to the extraction of the blood sample. The ethics committee of the Alava University Hospital, Spain, approved this study. All patients gave their written consent to participate in the study, which was conducted according to the provisions of the Helsinki declaration. After written informed consent was obtained, venous blood samples (10 mL) were collected from the volunteers, after which all the mood scales and cognitive tests were performed.

**Table 1:** **Demographic data (mean and SD for each group)**.

	HC	AD	LOBD
M/F	15/11	20/17	9/23
Age	72.81 ± 8.70	78.70 ± 5.86	68.88 ± 8.61
Education (0–5)	3.92 ± 1.14	3.33 ± 1.00	3.29 ± 1.14

### 2.2 Variable Description

For each subject in the study, we have measured the following 3 categories of variables.

#### 2.2.1 Neuropsychological Variables (NEURO)

Cognitive performance has been assessed with a battery of neuropsychological tests covering the following cognitive domains: executive function, learning and memory, and attention. The index for each cognitive domain is the mean of the z-scores of the tests covering that domain.

- Executive function: the executive function domain was assessed by combining measures from the following tests: Wisconsin Card Sorting Test (WCST) (Heaton, 1981; Spreen and Strauss, 1998), Stroop Test-Interference (SCWT) (Golden, 1978), Trail Making Test part B (TMT-B) (Reitan and Wolfson, 1985; Spreen and Strauss, 1998), and Functioning Assessment Short Test (FAS) (aka Controlled Oral Word Association Test) (Benton and Hamsher, 1989).
- Learning and memory: Wechsler Memory Scale (WMS-III) (Wechsler, 1997a,b) measuring five index scores: Auditory Memory, Visual Memory, Visual Working Memory, Immediate Memory, and Delayed Memory.
- Attention: the attention domain was assessed by combining the scores of the Trail Making Test part A (TMT-A) (Reitan and Wolfson, 1985), Stroop Colour–Word Test (SCWT) (Golden, 1978), and Digitsforward (DigitSpansubtest) (Wechsler Adult Intelligence Scale) (WAIS-III) (Wechsler, 1997a) (Spanish version by TEA, 1999).

#### 2.2.2 Biological Markers (BIO)

Selected biological markers for analysis were reported as relevant to LOBD and AD in published studies on inflammation based pathology (Akiyama et al., 2000; Kamer et al., 2008; Goldstein et al., 2009; Sardi et al., 2011; Lee et al., 2013; García-Bueno et al., 2014). After extracting plasma from blood samples, inflammatory cytokines interleukins 1 and 6 (IL-1 and IL-6) and tumor necrosis factor alpha (TNFα) were determined by enzyme immunoassay (EIA). Oxido-nitrosative parameters [nitrites and malondialdehyde (MDA)] were also analyzed in plasma samples. Cytokine levels were measured by EIA using reagents in kit form for TNFα (cat. 589201), Interleukin-1β (cat. 583311), and Interleukin 6 (cat. 501030) from Cayman Chemical Europe, Tallinn, Estonia. Plasma levels of TNFα, IL1β, and IL6 were measured in a 96-well plate and read at 405 nm following the manufacturer’s instructions. Nitrites (NO_2_), the final and stable product of nitric oxide, were measured using the Griess method, where samples are incubated in acidic solution with sulfanilamide and *N*-(1-naphthyl) ethylenediamine dihydrochloride (NEDA). The nitrites are converted into a pink compound that is measured photometrically at 540 nm (Synergy 2, Biotek). Lipid peroxidation, the final product of the reaction of oxido-nitrosative molecules with lipidic components of cells, was determined by Thiobarbituric Acid Reactive Substances (TBARS) assay (Cayman Chemical Europe, Tallinn, Estonia), based on the reaction of malondialdehyde (MDA) and thiobarbituric acid (TBA) under high temperature (95°C) and acidic conditions. The MDA-TBA adduct formed is measured colorimetrically at 530–540 nm (Synergy 2, Biotek). Plasma BDNF levels were measured using a BDNF Sandwich ELISA Kit, according to the manufacturer’s instructions (Millipore, USA, Cat. No. CYT306). Serum NGF levels were measured with an enzyme-linked immunosorbent assay (ELISA) method according to the manufacturer’s instructions, using a ChemiKineTM NGF Sandwich ELISA Kit (Millipore, USA, Cat No CYT304). All samples were assayed in duplicate. All plasma NF levels are expressed as picograms per milliliter.

#### 2.2.3 Clinical Observations (CLIN)

The Neuropsychiatric Inventory (NPI)^2^ (Cummings, 1997) was developed to provide a means of assessing neuropsychiatric symptoms and psychopathology of patients with Alzheimer’s disease and other neurodegenerative disorders. The NPI assesses 10 (10-item NPI) or 12 (2-item NPI) behavioral domains common in dementia. These include Hallucinations, Delusions, Agitation/aggression, Dysphoria/depression, Anxiety, Irritability, Disinhibition, Euphoria, Apathy, Aberrant motor behavior, Sleep, and night-time behavior change (12-item version only), Appetite and eating change (12-item version only). Each NPI domain is scored based on a standardized interview administered by the clinician for frequency, severity, and associated caregiver distress.

### 2.3 Functional Assessment

Patients were functionally assessed by the Functional Assessment Staging procedure (FAST) (Reisberg, 1988). Patients with greater functional impairment show increments in cognitive loss. FAST ranks patients in 16 stages. Stage 1 marks subjects without difficulties, while Stage 7(f) marks patients unable to hold up his/her head. The last eleven stages are subdivisions of FAST between the late stages 6 and 7. FAST was administered by the clinician leading the study.

### 2.4 Population Comparison

We used the Welch’s *t*-test to assess the statistical significance of variables differences between groups. It is a two-sample test used to check the hypothesis that two populations have equal means. Welch’s *t*-test is the adaptation of Student’s *t*-test for the case of two population samples may have different variances. These tests are often referred to as “unpaired” or “independent samples” *t*-tests, as they are typically applied when the statistical units underlying the two samples being compared are non-overlapping.

### 2.5 Classification Algorithms

Classification experiments were carried out in the Python programming language, using specific classifier implementations provided by scikit-learn^3^ (see text footnote 4) python package. We have applied the Support Vector Machines (SVM) (Vapnik, 1998), CART Decision Trees, and Random Forest (RF). Briefly described, SVM build a discriminating function with optimal generalization properties, which is a hyperplane built on the basis of the support vectors at the boundaries between classes. The kernel trick [e.g., radial basis function (RBF)] allows to deal with not linearly separable classes. Parameter tuning (i.e., Gaussian function width) is performed independently at each cross-validation fold when carrying out an assessment of classifier performance. SVM and its libSVM^4^ implementation (Chang and Lin, 2011) have become a standard classifier in the neuroscience community (Burges, 1998; Tao et al., 2006; Fung and Stoeckel, 2007). CART Decision Trees (Breiman et al., 1984; Quinlan, 1993) are built by recursive data space partitions. A univariate (single attribute) split is defined at each tree node using some criterion (e.g., mutual information, gain-ratio, impurity gini index). Tree leaves correspond to class *a posteriori* distribution of the training data samples falling in this leave. Random Forests (RF) (Breiman, 2001) algorithm is an ensemble of classifiers, which has been successfully applied in a wide variety of classification tasks (Barandiaran et al., 2010). RF is a collection of decorrelated randomly generated decision tree predictors, in which each tree casts a unit vote to decide the most popular class of input x. A bootstrapped training dataset is used to grow each individual tree. RF model parameters are the number of trees, their maximum depth, and the ratio of dimensionality reduction at each node. Finally, to assess variable importance, we have computed for each variable the average Gini impurity index (Breiman et al., 1984) of all the nodes in a CART classifier where this variable is used for the split, normalizing it such that the most important variable has value 1.

#### 2.5.1 Experimental Design

All variables are normalized computing their z-scores previous to classification experiments. In order to reduce circularity effects, variable normalization was carried out independently at each cross-validation folder. In order to evaluate the effect of neuropshycological measures (NEURO), biological markers (BIO), and clinical variables (CLIN) in differentiating HC from individuals with AD and LOBD, we have applied multivariate machine learning classification methods to each of the possible contrasts in the study: (1) healthy controls versus Alzheimer’s disease patients (HC vs. AD), (2) healthy controls versus Bipolar disorder patients (HC vs. LOBD), and (3) Bipolar disorder versus Alzheimer’s disease patients (LOBD vs. AD). We evaluated the performance of the classifier using a leave-one-out (LOO) cross-validation algorithm, applying a 3 × 2-fold cross-validation grid search for classifier optimal parameters tuning. To quantify the results, we measured the following performance measures: (a) accuracy ((TP + TN)/N), (b) specificity [TN/(FP + TN)], (c) sensitivity [TP/(TP + FN)], and (d) area under the ROC curve (AUC) (Faraggi and Reiser, 2002; Joachims, 2005), where TP is the number of true positives; TN is the number of true negatives; FP is the number of false positives; and FN is the number of false negatives.

## 3 Results

### 3.1 Statistical Group Comparison

Table 2 shows the Welch’s *t*-test *p*-values for the clinical, biological, and neuropsychological variables, respectively, for each of the possible contrasts. Entries highlighted in gray correspond to statistically significant differences. Most of the clinical variables have significant differences between groups, except Delusions and Anxiety. Euphoria differences are significant in HC vs. LOBD. Comparing LOBD vs. AD, only FAST and Agitation provide significant differences. The biological biomarkers, on the other hand, do not have any significant differences, with the exception of the MDA and TNFα in the case of LOBD vs. AD. Neuropsychological variables are statistically significant in the comparisons with controls, but in the comparison LOBD vs. AD, only the memory tests have significant differences.

**Table 2:** **Welch’s *t*-test *p*-value for each behavioral, biological biomarker, and aggregate neuropsychological variable (rows) *per* group contrast (columns)**.

	HC vs. AD	HC vs. LOBD	LOBD vs. AD
**CLINICAL**			
FAST	<0.001	<0.001	<0.001
TD1	0.022	0.045	0.871
TA1	0.009	<0.001	0.002
TDD	0.002	<0.001	0.145
TA2	0.002	<0.001	0.110
TE	0.325	0.008	0.026
TA3	<0.001	<0.001	0.579
TD2	0.065	0.356	0.618
TI	0.003	0.001	0.068
TC	0.003	0.096	0.230
TS	0.001	0.004	0.088

**BIOLOGICAL BIOMARKER**			
BDNF	0.631	0.263	0.425
NGF	0.090	0.916	0.074
NO_2_	0.468	0.233	0.763
TNFα	0.087	0.691	0.021
IL6	0.646	0.147	0.170
IL1	0.539	0.781	0.477
MDA	0.304	0.052	<0.001
**NEUROPSY**			
EF	<0.001	<0.001	0.337
A	<0.001	<0.001	0.804
M	<0.001	<0.001	<0.001

*Statistically significant entries (p < 0.01) are highlighted in gray*.

*TD1, total delusions; TA1, total agitation; TDD, total dysphoria/depression; TA2, total anxiety; TE, total euphoria; TA3, total apathy; TD2, total disinhibition; TI, total irritability; TC, total CMA; TS, total sleep; EF, executive functions; A, attention; M, memory*.

### 3.2 Variable Importance

**Figure 1:**
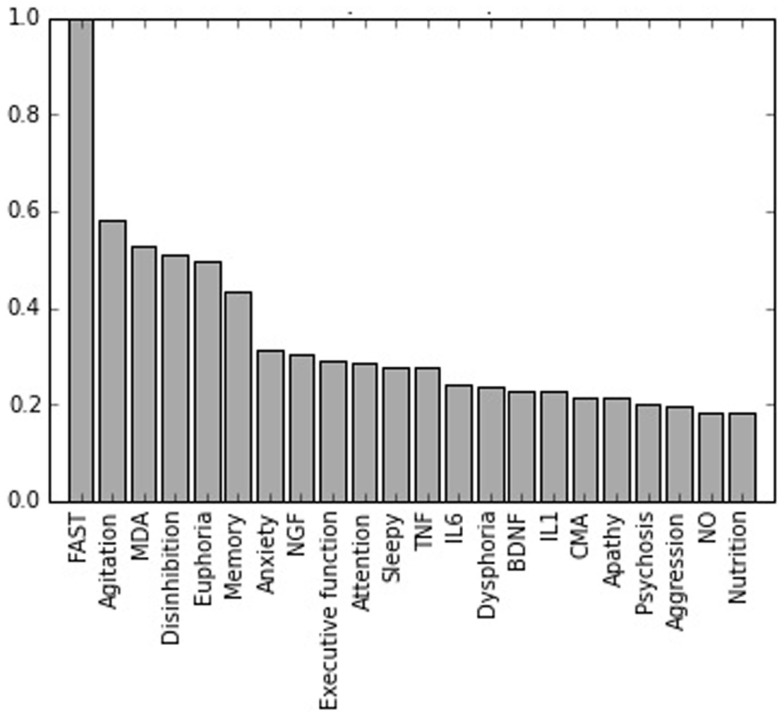
**Normalized feature importances in the BD vs. AD classification experiment**.

Figure 1 shows a plot of the feature importance of the variables considered in the study for the LOBD vs. AD classification contrast. The most important variable in all experiments is FAST. Overall, the feature importance values are in agreement with the statistically significant differences presented in Table 2, providing a more precise ranking. Cognitive tests are the second most important feature in the discrimination of HC vs. AD, whereas the clinical are more important discriminating HC vs. LOBD, with memory domain ranking high. For the critical discrimination of LOBD vs. AD, the clinical variables are the most informative; however, biological marker MDA ranks third while cognitive memory domain ranks fifth. This is the only instance of high importance ranking biological marker.

### 3.3 Classification

Classification performance results are presented in Tables 3 and 4 reporting accuracy, and AUC, respectively, for each classifier, combination of variables and classification contrast. Comparing classifier results, the CART provides the best results, though the improvement relative to other classifiers does not achieve statistical significance (*F*-test, *p* > 0.01). This may be due to the fact that small sample size penalizes the construction of large classifiers, which are overparameterized. Comparison of results according to variable category shows that clinical variables (CLIN) provide the best results or contribute to them. On the other hand, the biological biomarkers (BIO) are the ones that contribute less to classification performance. Considering the contrast LOBD vs. AD, the clinical variables provide the best results, though for some classifiers, such as RBF SVM, the neuropsychological variables contribute to improve results. The best results are obtained using the set of variables selected according to their significance in a Welch’s *t*-test (denoted CLIN + NEURO + BIO-Wt in the tables).

**Table 3:** **LOO accuracy estimation of the classifiers on the various combinations of features for each classification contrast**.

		CART	RF	SVM (rbf)	SVM (lin)
LOBD vs. AD	1. BIO	46.38	59.42	71.01	60.87
	2. NEURO	66.67	71.01	68.12	55.07
	3. BEHAV	85.51	84.06	79.71	84.06
	4. BIO + NEURO	66.67	62.32	71.01	66.67
	5. BIO + BEHAV	79.71	73.91	79.71	79.71
	6. NEURO + BEHAV	79.71	81.16	82.61	84.06
	7. NEURO + BEHAV + BIO	78.26	79.71	84.06	82.61
	8. NEURO + BEHAV + BIO-Wt	**90.26**	**86.67**	**89.06**	**87.51**
HC vs. AD	1. BIO	71.43	60.32	57.14	49.21
	2. NEURO	88.89	93.65	87.30	84.13
	3. BEHAV	95.24	95.24	93.65	95.24
	4. BIO + NEURO	85.71	87.30	87.30	88.89
	5. BIO + BEHAV	92.06	92.06	96.83	93.65
	6. NEURO + BEHAV	93.65	96.83	95.24	96.83
	7. NEURO + BEHAV + BIO	95.24	95.24	95.24	92.06
	8. NEURO + BEHAV + BIO-Wt	**96.24**	**97.21**	**97.21**	**97.21**
HC vs. LOBD	1. BIO	46.55	48.28	53.45	60.34
	2. NEURO	75.86	77.59	79.31	82.76
	3. BEHAV	89.66	89.66	91.38	91.38
	4. BIO + NEURO	79.31	82.76	77.59	77.59
	5. BIO + BEHAV	82.76	93.10	91.38	91.38
	6. NEURO + BEHAV	82.76	94.83	93.10	93.10
	7. NEURO + BEHAV + BIO	82.76	89.66	93.10	91.38
	8. NEURO + BEHAV + BIO-Wt	**91.76**	**95.76**	**94.10**	**94.38**

*Bold values are the maximum attained by a classifier *per* classification contrast*.

*BIO, blood biomarkers; BEHAV, clinical variables; NEURO, neuropsychological tests; Wt variables are selected according to Welch’s *t*-test*.

**Table 4:** **LOO AUC estimation of the classifiers on the various combinations of features for each classification contrast**.

		CART	RF	SVM (rbf)	SVM (lin)
LOBD vs. AD	1. BIO	45.78	59.21	71.28	61.19
	2. NEURO	66.60	71.28	68.58	55.15
	3. BEHAV	85.64	83.87	79.81	83.87
	4. BIO + NEURO	66.39	61.91	70.86	66.60
	5. BIO + BEHAV	79.81	73.35	80.03	79.81
	6. NEURO + BEHAV	80.24	80.74	82.73	84.08
	7. NEURO + BEHAV + BIO	78.67	79.39	84.50	82.73
	8. NEURO + BEHAV + BIO-Wt	**89.57**	**86.39**	**88.25**	**87.33**
HC vs. AD	1. BIO	70.53	57.07	55.51	47.61
	2. NEURO	88.25	92.88	87.47	84.20
	3. BEHAV	95.37	95.37	94.02	94.80
	4. BIO + NEURO	84.98	87.47	87.47	88.83
	5. BIO + BEHAV	92.10	91.53	96.73	93.45
	6. NEURO + BEHAV	93.45	96.73	95.37	96.15
	7. NEURO + BEHAV + BIO	95.37	95.37	95.95	92.67
	8. NEURO + BEHAV + BIO-Wt	**97.33**	**98.17**	**98.17**	**97.77**
HC vs. LOBD	1. BIO	45.79	47.72	53.12	59.01
	2. NEURO	76.32	77.16	79.45	82.93
	3. BEHAV	89.54	89.90	91.47	91.11
	4. BIO + NEURO	79.81	82.57	77.88	77.88
	5. BIO + BEHAV	82.57	93.03	91.47	91.47
	6. NEURO + BEHAV	82.93	94.23	93.75	93.39
	7. NEURO + BEHAV + BIO	82.57	89.18	93.75	91.83
	7. NEURO + BEHAV + BIO-Wt	**91.37**	**96.38**	**95.55**	**95.55**

*Bold values are the maximum attained by a classifier *per* classification contrast*.

*BIO, blood biomarkers; BEHAV, clinical variables; NEURO, neuropsychological tests; Wt variables selected according to Welch’s *t*-test*.

## 4 Discussion

This study was designed to investigate the feasibility of discriminating between AD and LOBD (Lebert et al., 2008; Carlino et al., 2013; Grande et al., 2014) using a wide range of clinical, neuropsychological, and biological (inflammatory, oxido-nitrosative, and neurotrophic) measures for this purpose. Previous studies have attempted to discriminate between subjects with AD and HC and between LOBD and HC, but there is no other study to our knowledge dealing with LOBD patients compared with AD, when the differential diagnosis is more difficult (Aprahamian et al., 2014). We have included HC vs. AD and HC vs. LOBD contrasts in Tables 2–4 to assess if the biomarkers are also useful to discriminate them. We find that the results obtained are according to the literature. We have found that the clinical variables carry most of the diagnostic value; however, classification performance can be improved by the consideration of the neuropsychological variables and biological markers.

### 4.1 Clinical Variables

It has been observed that cognitive deficits affect the functionality and global prognosis of LOBD patients (Kawas et al., 2003) as occurs in patients with dementia. Besides cognitive performance, behavioral disorders are also closely related to the overall functionality of the patients. Non-cognitive symptoms have to be considered as they may help the discrimination between LOBD and AD. Our results in Figure 1 show that agitation, euphoria, and disinhibition are the non-cognitive neuropsychological variables having the greatest discrimination power in the case AD vs. LOBD. Nevertheless, the clinical variable that differentiates more strongly between LOBD and AD is overall patient behavioral functionality measured by FAST.

### 4.2 Neuropsychological Variables

Neuropsychological assessment is typically used for both descriptive and diagnostic purposes. When used diagnostically, tests provide information about how likely is that a particular individual has or will have a cognitive disorder. In relation to BD, various studies have revealed cognitive impairment as part of its clinical expression. In fact, some authors suggested that having been diagnosed with BD is a significant predictor of cognitive decline over time, further, cognitive dysfunction increases in the long term (Lewandowski et al., 2011; Torrent et al., 2012). Although there are limited data on the cognitive profile of LOBD (Carlino et al., 2013; Grande et al., 2014), cognitive deficits affecting memory, attention, and executive function have been reported (Robinson et al., 2006; Osher et al., 2011; Aprahamian et al., 2014). Accordingly, when comparing LOBD patients with HC we found that all these variables have statistically significant differences, as shown in Table 2. Similar cognitive degradation is well known in AD (Kawas et al., 2003; Albert and Blacker, 2006), and it is confirmed by results in Table 2. The classification experiments confirm that the set of neuropsychological variables (NEURO) is useful to discriminate AD patients from controls, achieving high accuracy (93.65%) and AUC (92.88%). However, they are much less effective to discriminate LOBD patients from controls and AD patients. The results of Welch’s *t*-test in Table 2 and the variable importance results in Figure 1 confirm that memory cognitive domain is essential in clinical practice for the detection and diagnosis of AD (Weintraub et al., 2012). Accordingly, Figure 1 shows that memory domain tests have a high importance for AD vs. LOBD classification. Finally, we found that memory is a key factor in differential diagnosis between LOBD and AD.

### 4.3 Blood Biomarkers

Besides the similarity of some symptoms, AD and LOBD share pathophysiological features that might difficult differential diagnosis. Peripheral markers related to inflammation, oxidative stress, and neurotrophins have been related to clinical symptoms, cognitive decline, and illness severity in BD (Barbosa et al., 2012; Martinez-Cengotitabengoa et al., 2014), as well as in AD (Berridge, 2013). In our study, all blood biomarkers, except IL1, were lower in the plasma of LOBD group than in AD group, although only MDA levels revealed statistical significant difference in the Welch’s *t*-test. This finding agrees with a significant decrease in BDNF and IL-6 in BD patients at later stage compared to its early stage, while, inversely, TNFα has a significant increase at the BD later stage (Kauer-Sant’Anna et al., 2009; Grande et al., 2014). All these findings may suggest that the group of LOBD patients have more inflammation. No discriminant variable has been found from the collection of biological biomarkers (BIO) in our classification experiments. There are numerous reports of inflammation and excess oxidation within the brain of patients, but outside the CNS, the evidence is less definite and results of studies are often contradictory. It has been suggested that inflammation and oxidative stress do not cause AD or LOBD by themselves, but probably during aging, they reinforce many interdependent factors related to these complex neuropsychiatric disorders (Forcada et al., 2014). It is well known that brain aging involves complex structural and molecular processes that provide a misbalance between protective and degenerative factors, predisposing the brain to higher risk of acquiring neurodegenerative diseases (Lewandowski et al., 2011). Nevertheless, the inclusion of MDA in the Welch’s *t*-test features produces a great improvement in classification performance, reaching accuracy 90.26 and AUC 89.57. This is a surprising fact because inflammation is a common effect not a differential effect.

### 4.4 Limitations

The sample is not well balanced; there are diverse numbers of AD, LOBD, and HC. The feminine LOBD sample is much larger. Old age patients suffer from multimorbidity, which is a source of confusion for blood plasma biomarkers.

## 5 Conclusion

We have found that a small set of variables, including an oxidative stress biomarker (i.e., MDA), allows good discrimination of LOBD and AD. Besides the potential construction of a CAD system upon larger databases, these findings could help in identifying new therapeutic routes for treatment and diagnosis.

## Author Contributions

AB, IG, AG-P, and MG have made substantial contributions to the conception or design of the work; AB, JM, JL, DC, BA, AS, MG, and EE contributed to the acquisition, analysis, or interpretation of data for the work; all authors contributed in drafting the work and revising it critically for important intellectual content; all authors gave final approval of the version to be published; all authors agreed to be accountable for all aspects of the work in ensuring that questions related to the accuracy or integrity of any part of the work are appropriately investigated and resolved.

## Conflict of Interest Statement

The authors declare that the research was conducted in the absence of any commercial or financial relationships that could be construed as a potential conflict of interest.

## Footnotes

^1^http://www.controlled-trials.com/search?q=HS_2FPI2010001

^2^http://www.dementia-assessment.com.au/behavioural/

^3^http://scikit-learn.org/stable/

^4^http://www.csie.ntu.edu.tw/~cjlin/libsvm/

## References

[B1] Akiyama H. Barger S. Barnum S. Bradt B. Bauer J. Cole G. M. (2000). Inflammation and Alzheimer’s disease. Neurobiol. Aging 21, 383–421. 10.1016/S0197-4580(00)00124-X 10858586 PMC3887148

[B2] Albert M. S. Blacker D. (2006). Mild cognitive impairment and dementia. Annu. Rev. Clin. Psychol. 2, 379–388. 10.1146/annurev.clinpsy.1.102803.144039 17716075

[B3] Aprahamian I. Ladeira R. M. Diniz B. S. Forlenza O. V. Nunes P. V. (2014). Cognitive impairment in euthymic older adults with bipolar disorder: a controlled study using cognitive screening tests. Am. J. Geriatr. Psychiatry 22, 389–397. 10.1016/j.jagp.2012.08.013 23567429

[B4] Azorin J. M. Belzeaux R. Adida M. (2015). Age-at-onset and comorbidity may separate depressive disorder subtypes along a descending gradient of bipolar propensity. Behav. Brain Res. 282, 185–193. 10.1016/j.bbr.2015.01.014 25596332

[B5] Barandiaran I. Paloc C. Graña M. (2010). Real-time optical markerles tracking for augmented reality applications. J. Real Time Image Process. 5, 129–138. 10.1007/s11554-009-0140-2

[B6] Barbosa I. G. Rocha N. P. Huguet R. B. Ferreira R. A. Salgado J. V. Carvalho L. A. (2012). Executive dysfunction in euthymic bipolar disorder patients and its association with plasma biomarkers. J. Affect. Disord. 137, 151–155. 10.1016/j.jad.2011.12.034 22252095

[B7] Bauer I. E. Pascoe M. C. Wollenhaupt-Aguiar B. Kapczinski F. Soares J. C. (2014a). Inflammatory mediators of cognitive impairment in bipolar disorder. J. Psychiatr. Res. 56, 18–27. 10.1016/j.jpsychires.2014.04.017 24862657 PMC4167370

[B8] Bauer M. Glenn T. Alda M. Andreassen O. A. Angelopoulos E. Ardau R. (2014b). Relationship between sunlight and the age of onset of bipolar disorder: an international multisite study. J. Affect. Disord. 167, 104–111. 10.1016/j.jad.2014.05.032 24953482

[B9] Bauer M. Glenn T. Alda M. Andreassen O. A. Angelopoulos E. Ardau R. (2015a). Influence of birth cohort on age of onset cluster analysis in bipolar I disorder. Eur. Psychiatry 30, 99–105. 10.1016/j.eurpsy.2014.10.005 25498240

[B10] Bauer M. Glenn T. Alda M. Andreassen O. A. Angelopoulos E. Ardau R. (2015b). Influence of light exposure during early life on the age of onset of bipolar disorder. J. Psychiatr. Res. 64, 1–8. 10.1016/j.jpsychires.2015.03.013 25862378

[B11] Benton A. L. Hamsher K. (1989). Multilingual Aphasia Examination. Iowa City, IA: University of Iowa.

[B12] Berridge M. J. (2013). Dysregulation of neural calcium signaling in Alzheimer disease, bipolar disorder and schizophrenia. Prion 7, 2–13. 10.4161/pri.21767 22895098 PMC3609045

[B13] Besga A. Martinez-Cengotitabengoa M. Gonzalez-Ortega I. Gutierrez M. Barbeito S. Gonzalez-Pinto A. (2011). The role of white matter damage in late onset bipolar disorder. Maturitas 70, 160–163. 10.1016/j.maturitas.2011.07.005 21872409

[B14] Besga A. Termenon M. Graña M. Echeveste J. Perez J. Gonzalez-Pinto A. (2012). Discovering Alzheimer’s disease and bipolar disorder white matter effects building computer aided diagnostic systems on brain diffusion tensor imaging features. Neurosci. Lett. 520, 71–76. 10.1016/j.neulet.2012.05.033 22617636

[B15] Breiman L. (2001). Random forests. Mach. Learn. 45, 5–32. 10.1023/A:1017934522171

[B16] Breiman L. Friedman J. Olshen R. Stone C. (1984). Classification and Regression Trees. Monterey, CA: Wadsworth and Brooks.

[B17] Brydon L. Walker C. Wawrzyniak A. Whitehead D. Okamura H. Yajima J. (2009). Synergistic effects of psychological and immune stressors on inflammatory cytokine and sickness responses in humans. Brain Behav. Immun. 23, 217–224. 10.1016/j.bbi.2008.09.007 18835437 PMC2637301

[B18] Burges C. (1998). A tutorial on support vector machines for pattern recognition. Data Min. Knowl. Discov. 2, 121–167. 10.1023/A:1009715923555

[B19] Carlino A. R. Stinnett J. L. Kim D. R. (2013). New onset of bipolar disorder in late life. Psychosomatics 54, 94–97. 10.1016/j.psym.2012.01.006 22652303 PMC3914401

[B20] Castanon N. Lasselin J. Capuron L. (2014). Neuropsychiatric comorbidity in obesity: role of inflammatory processes. Front. Endocrinol. 5:74. 10.3389/fendo.2014.00074 24860551 PMC4030152

[B21] Chang C. C. Lin C. J. (2011). Libsvm: a library for support vector machines. ACM Trans. Intell. Syst. Technol. 2, 27 10.1145/1961189.1961199

[B22] Cummings J. (1997). The neuropsychiatric inventory: assessing psychopathology in dementia patients. Neurology 48(Suppl. 6), S10–S16. 10.1212/WNL.48.5_Suppl_6.10S 9153155

[B23] Depp C. Jeste D. (2004). Bipolar disorder in older adults: a critical review. Bipolar Disord. 6, 343–367. 10.1111/j.1399-5618.2004.00139.x 15383127

[B24] Dickerson F. Stallings C. Origoni A. Vaughan C. Katsafanas E. Khushalani S. (2013). A combined marker of inflammation in individuals with mania. PLoS ONE 8:e73520. 10.1371/journal.pone.0073520 24019926 PMC3760815

[B25] Faraggi D. Reiser B. (2002). Estimation of the area under the roc curve. Stat. Med. 21, 3093–3106. 10.1002/sim.1228 12369084

[B26] Forcada I. Mur M. Mora E. Vieta E. Bartras-Faz D. Portella M. J. (2014). The influence of cognitive reserve on psychosocial and neuropsychological functioning in bipolar disorder. Eur. Neuropsychopharmacol. 25, 214–222. 10.1016/j.euroneuro.2014.07.018 25172270

[B27] Fung G. Stoeckel J. (2007). Svm feature selection for classification of spect images of Alzheimer’s disease using spatial information. Knowl. Inf. Syst. 11, 243–258. 10.1007/s10115-006-0043-5

[B28] García-Bueno B. Bioque M. Mac-Dowell K. S. Barcones M. F. Martínez-Cengotitabengoa M. Pina-Camacho L. (2014). Proanti-inflammatory dysregulation in patients with first episode of psychosis: toward an integrative inflammatory hypothesis of schizophrenia. Schizophr. Bull. 40, 376–387. 10.1093/schbul/sbt001 23486748 PMC3932081

[B29] Golden C. (1978). The Stroop Color and Word Test: A Manual for Clinical and Experimental Uses. Chicago, IL: Stoelting Co.

[B30] Goldstein B. Kemp D. Soczynska J. McIntyre R. (2009). Inflammation and the phenomenology, pathophysiology, comorbidity, and treatment of bipolar disorder: a systematic review of the literature. J. Clin. Psychiatry 70, 1078–1090. 10.4088/JCP.08r04505 19497250

[B31] Graña M. Termenon M. Savio A. Gonzalez-Pinto A. Echeveste J. Perez J. (2011). Computer aided diagnosis system for Alzheimer disease using brain diffusion tensor imaging features selected by Pearson’s correlation. Neurosci. Lett. 502, 225–229. 10.1016/j.neulet.2011.07.049 21839143

[B32] Grande I. Magalhaes P. V. Chendo I. Stertz L. Panizutti B. Colpo G. D. (2014). Staging bipolar disorder: clinical, biochemical, and functional correlates. Acta Psychiatr. Scand. 129, 437–444. 10.1111/acps.12268 24628576

[B33] Heaton R. K. (1981). Wisconsin Card Sorting Test Manual. Odessa, FL: Psychological Assessment Resources.

[B34] Hope S. Hoseth E. Dieset I. Mørch R. H. Aas M. Aukrust P. (2015). Inflammatory markers are associated with general cognitive abilities in schizophrenia and bipolar disorder patients and healthy controls. Schizophr. Res. 165, 188–194. 10.1016/j.schres.2015.04.004 25956633

[B35] Joachims T. (2005). “A support vector method for multivariate performance measures,” in Proceedings of the 22nd International Conference on Machine Learning, (Edinburgh), 377–384.

[B36] Kamer A. R. Craig R. G. Dasanayake A. P. Brys M. Glodzik-Sobanska L. de Leon M. J. (2008). Inflammation and Alzheimer’s disease: possible role of periodontal diseases. Alzheimers Dement. 4, 242–250. 10.1016/j.jalz.2007.08.004 18631974

[B37] Kauer-Sant’Anna M. Kapczinski F. Andreazza A. C. Bond D. J. Lam R. W. Young L. T. (2009). Brain-derived neurotrophic factor and inflammatory markers in patients with early- vs. late-stage bipolar disorder. Int. J. Neuropsychopharmacol. 12, 447–458. 10.1017/S1461145708009310 18771602

[B38] Kawas C. H. Corrada M. M. Brookmeyer R. Morrison A. Resnick S. M. Zonderman A. B. (2003). Visual memory predicts Alzheimer’s disease more than a decade before diagnosis. Neurology 60, 1089–1093. 10.1212/01.WNL.0000055813.36504.BF 12682311

[B39] Konradi C. Sillivan S. E. Clay H. B. (2012). Mitochondria, oligodendrocytes and inflammation in bipolar disorder: evidence from transcriptome studies points to intriguing parallels with multiple sclerosis. Neurobiol. Dis. 45, 37–47. 10.1016/j.nbd.2011.01.025 21310238 PMC3117935

[B40] Lebert F. Lys H. Haem E. Pasquier F. (2008). Dementia following bipolar disorder. Encephale 34, 606–610. 10.1016/j.encep.2007.12.007 19081458

[B41] Leboyer M. Soreca I. Scott J. Frye M. Henry C. Tamouza R. (2012). Can bipolar disorder be viewed as a multi-system inflammatory disease? J. Affect. Disord. 141, 1–10. 10.1016/j.jad.2011.12.049 22497876 PMC3498820

[B42] Lee S.-Y. Chen S.-L. Chang Y.-H. Chen P. Huang S.-Y. (2013). Inflammation’s association with metabolic profiles before and after a twelve-week clinical trial in drug-naive patients with bipolar ii disorder. PLoS ONE 8:e66847 10.1371/journal.pone.0066847 23826157 PMC3695222

[B43] Lewandowski K. E. Cohen B. M. Ongur D. (2011). Evolution of neuropsychological dysfunction during the course of schizophrenia and bipolar disorder. Psychol. Med. 41, 225–241. 10.1017/S0033291710001042 20836900

[B44] Martinez-Cengotitabengoa M. Mico J. A. Arango C. Castro-Fornieles J. Graell M. Paya B. (2014). Basal low antioxidant capacity correlates with cognitive deficits in early onset psychosis. a 2-year follow-up study. Schizophr. Res. 156, 23–29. 10.1016/j.schres.2014.03.025 24768133

[B45] Ng B. Camacho A. Lara D. R. Brunstein M. G. Pinto O. C. Akiskal H. S. (2008). A case series on the hypothesized connection between dementia and bipolar spectrum disorders: bipolar type VI? J. Affect. Disord. 107, 307–315. 10.1016/j.jad.2007.08.018 17889374

[B46] Osher Y. Dobron A. Belmaker R. H. Bersudsky Y. Dwolatzky T. (2011). Computerized testing of neurocognitive function in euthymic bipolar patients compared to those with mild cognitive impairment and cognitively healthy controls. Psychother. Psychosom. 80, 298–303. 10.1159/000324508 21646824

[B47] Po-Han C. Wan-Ju T. Lin-Mei C. Chih-Chien L. Tsuo-Hung L. Chin-Hong C. (2015). Late onset bipolar disorder: a case report and review of the literature. J. Clin. Gerontol. Geriatr. 6, 27–29. 10.1016/j.jcgg.2014.05.002

[B48] Prabhakar D. Balon R. (2010). Late-onset bipolar disorder a case for careful appraisal. Psychiatry (Edgmont) 7, 34–37. 20386635 PMC2848458

[B49] Quinlan J. R. (1993). C4.5: Programs for Machine Learning. San Mateo, CA: Morgan Kaufmann.

[B50] Ramirez J. Gorriz J. M. Segovia F. Chaves R. Salas-Gonzalez D. Lopez M. (2010). “Early Alzheimer’s disease diagnosis using partial least squares and random forests,” in IEEE International Symposium on Biomedical Imaging: From Nano to Macro (Rotterdam: IEEE), 81–84.

[B51] Rao J. S. Harry G. J. Rapoport S. I. Kim H. W. (2010). Increased excitotoxicity and neuroinflammatory markers in postmortem frontal cortex from bipolar disorder patients. Mol. Psychiatry 15, 384–392. 10.1038/mp.2009.47 19488045 PMC2844920

[B52] Rao J. S. Keleshian V. L. Klein S. Rapoport S. I. (2012). Epigenetic modifications in frontal cortex from Alzheimer’s disease and bipolar disorder patients. Transl. Psychiatry 2, e132 10.1038/tp.2012.55 22760556 PMC3410632

[B53] Rao J. S. Rapoport S. Kim H.-W. (2011). Altered neuroinflammatory, arachidonic acid cascade and synaptic markers in postmortem Alzheimer’s disease brain. Transl. Psychiatry 1, 1–9. 10.1038/tp.2011.27 PMC3309508 22832605

[B54] Reisberg B. (1988). Functional assessment staging (fast). Psychopharmacol. Bull. 24, 653–659. 3249767

[B55] Reitan R. M. Wolfson D. (1985). The Halstead-Reitan Neuropsycholgical Test Battery: Therapy and Clinical Interpretation. Tucson, AZ: Neuropsychological Press.

[B56] Robinson L. J. Thompson J. M. Gallagher P. Goswami U. Young A. H. (2006). A meta-analysis of cognitive deficits in euthymic patients with bipolar disorder. J. Affect. Disord. 93, 105–115. 10.1016/j.jad.2006.02.016 16677713

[B57] Salas-Gonzalez D. Górriz J. M. Ramírez J. López M. Illan I. A. Segovia F. (2009). Analysis of SPECT brain images for the diagnosis of Alzheimer’s disease using moments and support vector machines. Neurosci. Lett. 461, 60–64. 10.1016/j.neulet.2009.05.056 19477227

[B58] Sardi F. Fassina L. Venturini L. Inguscio M. Guerriero F. Rolfo E. (2011). Alzheimer’s disease, autoimmunity and inflammation. the good, the bad and the ugly. Autoimmun. Rev. 11, 149–153. 10.1016/j.autrev.2011.09.005 21996556

[B59] Savio A. Garcia-Sebastian M. Chyzyk D. Hernandez C. Graña M. Sistiaga A. (2011). Neurocognitive disorder detection based on feature vectors extracted from vbm analysis of structural MRI. Comput. Biol. Med. 41, 600–610. 10.1016/j.compbiomed.2011.05.010 21621760

[B60] Sigut J. Piñeiro J. Gonzalez E. Torres J. (2007). An expert system for supervised classifier design: application to Alzheimer diagnosis. Exp. Syst. Appl. 32, 927–938. 10.1016/j.eswa.2006.01.026

[B61] Singhal G. Jaehne E. J. Corrigan F. Toben C. Baune B. T. (2014). Inflammasomes in neuroinflammation and changes in brain function: a focused review. Front. Neurosci. 8:315. 10.3389/fnins.2014.00315 25339862 PMC4188030

[B62] Spreen O. Strauss E. (1998). A Compendium of Neuropsychological Tests: Administration, Norms and Commentary. New York, NY: Oxford University Press.

[B63] Takeshi T. Hideki N. Yoshiaki I. Tatsuya O. Jun N. Noboru I. (2006). Lithium and dementia: a preliminary study. Prog. Neuropsychopharmacol. Biol. Psychiatry 30, 1125–1128. 10.1016/j.pnpbp.2006.04.020 16753246

[B64] Tao D. Tang X. Li X. Wu X. (2006). Asymmetric bagging and random subspace for support vector machines-based relevance feedback in image retrieval. IEEE Trans. Pattern Anal. Mach. Intell. 28, 1088–1099. 10.1109/TPAMI.2006.134 16792098

[B65] Termenon M. Graña M. Besga A. Echeveste J. Gonzalez-Pinto A. (2013). Lattice independent component analysis feature selection on diffusion weighted imaging for Alzheimer’s disease classification. Neurocomputing 114, 132–141. 10.1016/j.neucom.2012.08.044

[B66] Torrent C. Martinez-Aran A. del Mar Bonnin C. Reinares M. Daban C. Sole B. (2012). Long-term outcome of cognitive impairment in bipolar disorder. J. Clin. Psychiatry 73, e899–e905. 10.4088/JCP.11m07471 22901360

[B67] Vapnik V. (1998). Statistical Learning Theory. Hoboken, NJ: Wiley-Interscience.

[B68] Wechsler D. (1997a). Wechsler Adult Intelligence Scale, (Administration and Scoring Manual) (WAIS-III), 3rd Edn San Antonio, TX: The Psychological Corporation.

[B69] Wechsler D. (1997b). Wechsler Memory Scale, Third Ed. (Technical Manual). San Antonio, TX: The Psychological Corporation.

[B70] Weintraub S. Wicklund A. H. Salmon D. P. (2012). The neuropsychological profile of Alzheimer disease. Cold Spring Harb. Perspect. Med. 2, a006171–a006171. 10.1101/cshperspect.a006171 22474609 PMC3312395

[B71] Westman E. Simmons A. Zhang Y. Muehlboeck J. S. Tunnard C. Liu Y. (2011). Multivariate analysis of MRI data for Alzheimer’s disease, mild cognitive impairment and healthy controls. Neuroimage 54, 1178–1187. 10.1016/j.neuroimage.2010.08.044 20800095

[B72] Zahodne L. B. Ornstein K. Cosentino S. Devanand D. P. Stern Y. (2015). Longitudinal relationships between Alzheimer disease progression and psychosis, depressed mood, and agitation/aggression. Am. J. Geriatr. Psychiatry 23, 130–140. 10.1016/j.jagp.2013.03.014 23871118 PMC3858495

